# Biphasic lipid extraction from microalgae after PEF-treatment reduces the energy demand of the downstream process

**DOI:** 10.1186/s13068-025-02608-7

**Published:** 2025-01-28

**Authors:** Ioannis Papachristou, Natalja Nazarova, Rüdiger Wüstner, Robin Lina, Wolfgang Frey, Aude Silve

**Affiliations:** Institute for Pulsed Power and Microwave Technology (IHM), Karlsruhe Institute of Technology (KIT), Eggenstein-Leopoldshafen, Germany

**Keywords:** Pulsed electric field, Biphasic lipid extraction, Microalgae

## Abstract

**Background:**

The gradual extrusion of water-soluble intracellular components (such as proteins) from microalgae after pulsed electric field (PEF) treatment is a well-documented phenomenon. This could be utilized in biorefinery applications with lipid extraction taking place after such an ‘incubation’ period, i.e., a post-PEF-treatment step during which the biomass is left undisturbed before any further processing. The goal of this work was to further explore how this incubation could improve lipid extraction.

**Results:**

Experiments were conducted on wet, freshly harvested *Auxenochlorella protothecoides*, treated with 0.25 or 1.5 MJ/kg_DW_ and incubated for 24 h. Lipid extraction took place with a monophasic ethanol:hexane:water, 1:0.41:0.04 vol/vol/vol mixture with a 75.6 mL solvent per 1 g of dry biomass ratio. The kinetics of the extraction were studied with samples taken between 10 and 1080 min from fresh and incubated biomass. The yields at 10 min were significantly increased with incubation compared to without (31.2% dry weight compared to 1.81%, respectively). The experimental data were fitted with the Patricelli model where extraction occurs in two steps, a rapid washing of immediate available lipids and a slower diffusion one. During Nile-Red staining of microalgae and microscopy imaging, a shift of emission from both GFP and RFP channels to mostly RFP was observed indicating an increase in the polarity of the environment of Nile-Red. These led to an adaption of a biphasic ethanol:hexane:water 1:6:0.4 vol/vol/vol solvent with 37 mL solvent per 1 g of dry biomass ratio which while ineffective on fresh biomass, achieved a 27% dry weight yield from incubated microalgae. The extraction efficiency in the biphasic route was lower compared to the monophasic (i.e., 69% and 95%, respectively). It was compensated however, by the significant solvent reduction (37 mL to 75.6 mL respectively), in particular the ethanol minimization. For the extraction of 1 L lipids, it was estimated that the energy consumption ratio for the biphasic process was 1.6 compared to 9.9 for monophasic, making clearly the most preferential one.

**Conclusions:**

This biphasic approach significantly reduces solvent consumption and the respective energy requirement for solvent recovery. Incubation thus could majorly improve the commercialization prospects of the process.

**Graphical abstract:**

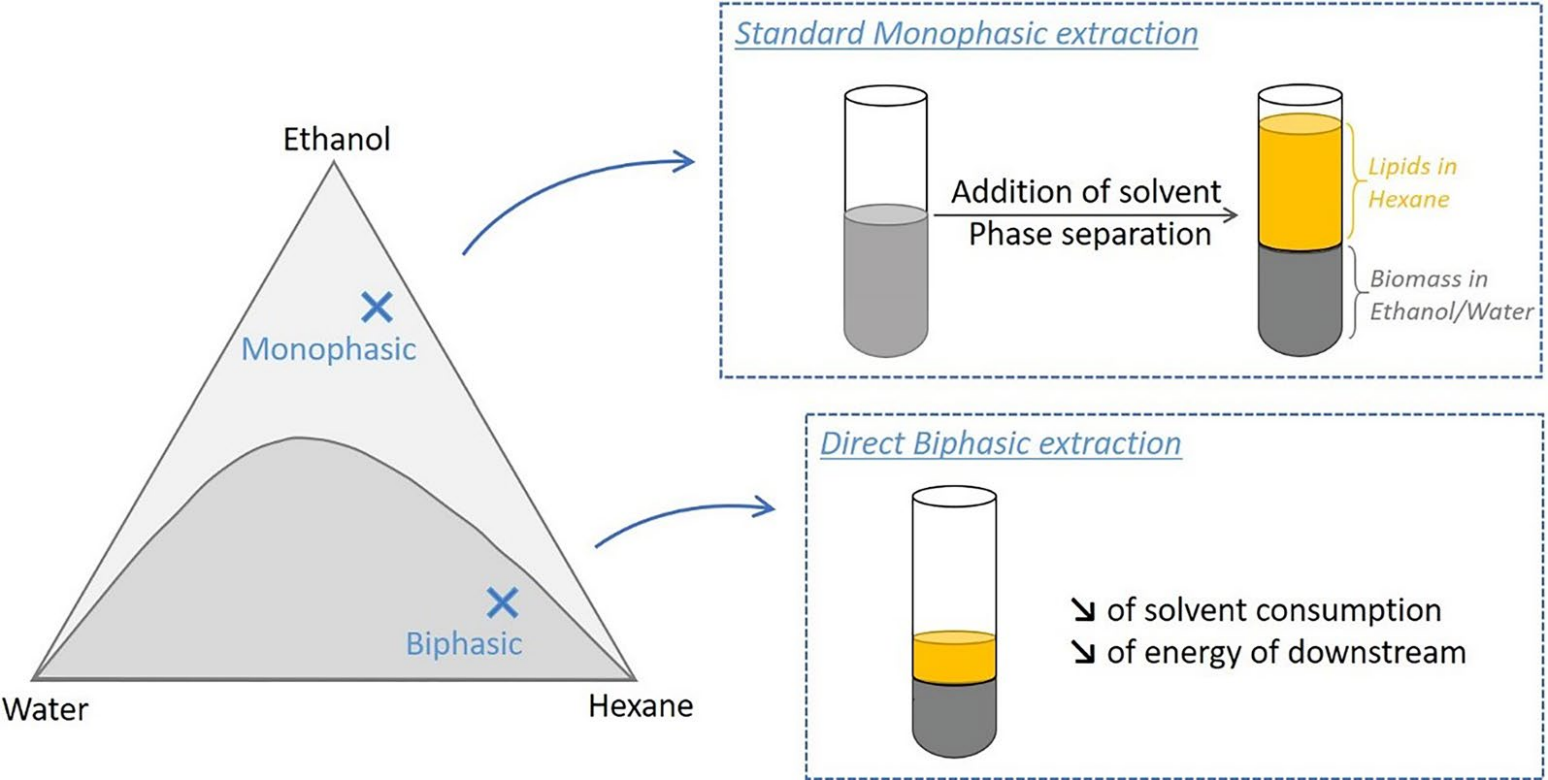

## Introduction

Microalgae are aquatic microscopic photosynthetic organisms, characterized by their remarkably diverse product output, high growth rates and ability to be cultivated in a diverse range of growth media [1] such wastewaters [2]. For these reasons, microalgae have been recognized as potential feedstocks in a number of fields, such as alternative biofuels [3], food ingredients and feed [4] and more niche markets such as healthcare products [5].

Lipids are one of the main constituents of microalgae, ranging between 15 and 50% of their mass depending on the cultivation conditions and strain [6]. They can be distinguished into polar and neutral lipids, based on their polarity. The former are typically building materials of the cell membranes, whereas the latter are accumulated inside the cell in the form of lipid droplets and serve as energy storage [7]. The lipid composition is an important factor which at the end determines for what applications the lipids are suitable. In general, it can be considered that microalgae with an overall high saturated lipid content in the form of triglycerides can be used for biodiesel production [8], whereas polyunsaturated fatty acids of the n-3 and n-6 families (PUFAs) are valuable as supplements for human diet or in aquacultures [9].

Most conventional lipid extraction techniques involve the use of organic solvents [10]. In simple terms, it involves mixing the microalgae with one or more solvents for an adequate time period in order to achieve the separation of lipids (‘solutes’) from the biomass matrix. As a rule, solutes dissolve better in solvents of similar polarity, a rule empirically known as ‘like dissolves like’ [11]. For an efficient extraction of both neutral and polar lipids, like the ones produced by microalgae, a mixture of polar and nonpolar solvents is thus often used. The Folch protocol and its later modification from Bligh and Dyer, were the first to apply this methodology for total lipid extraction from animal tissue utilizing a monophasic mixture of chloroform and methanol [12]. These solvents, while recognized as ‘gold standards’ for bench-scale extraction of lipids, are not suitable for larger-scale applications due to their toxicity [13]. A number of organic solvents have been examined to replace them such as hexane, heptane, ethyl acetate, acetone, ethanol and isopropanol [14, 15]. Hexane, ethanol and acetone are popular choices, due to the fact that their use is already accepted in food processing [16]. Hexane’s popularity is further reinforced by its highly neutral nature, low boiling point and low enthalpy of vaporization [17].

The microalgae cells are surrounded by a rigid cell wall which constitutes an obstacle to efficient lipid extraction. The cell walls are mainly composed of cellulose, hemicellulose and other polysaccharides [18] and have been recognized as a significant barrier for intracellular extraction from microalgae. Therefore, a pretreatment method is usually applied on the microalgae with the aim of conditioning the cell to enhance the accessibility to the targeted intracellular components [19]. This pretreatment can be physical (mechanical, thermal, electrical, etc.), chemical, biological or a combination of the above [20]. Pulsed electric fields (PEF) is one such physical pretreatment method [21, 22]. During PEF-treatment, the microalgae are subjected into an external electric field resulting in an increase of the transmembrane voltage. Once a voltage critical value is reached, the molecular structure of the cell membrane is re-arranged in a rapid progress leading to its destructuring and permeabilisation, a phenomenon also known as electroporation [23].

PEF is a mild technique, which does not destroy the cells into pieces and therefore does not generate debris, something which facilitates a smoother cascade extraction of different components from the microalgae [24, 25]. Moreover, once electroporation is achieved, a spontaneous release of intracellular water-soluble components to the surrounding aqueous solvent is reported [26, 27]. The above makes PEF particularly advantageous in a biorefinery scheme. In such applications, the entirety of the biomass is valorized with the extraction and separation of multiple products (examples of such approaches can be found in [28–32]). However, only a handful studies using PEF for cascade extractions from microalgae exist [25, 33–36].

In a previous work from our group, extraction of carbohydrates was achieved by subjecting PEF-treated *Auxenochlorella protothecoides* (*A. protothecoides*) to a 24-h spontaneous water extraction, followed by total lipid removal from the spent biomass using a monophasic ethanol:hexane:water blend [35]. Intriguingly, implementing that first water extraction step, henceforth referred to as ‘incubation’, made the lipid extraction more efficient in terms of yields and energy input compared to when it was performed directly after PEF-treatment of the microalgae. Moreover, in a previous publication from our group, it was demonstrated that the fatty acid methyl acid (FAMEs) composition of *A. protothecoides* was affected neither by the PEF-treatment, the intensity of treatment energy nor the incubation itself ([37]). Other works such as Luengo et al. [38] and Martínez et al. [39] similarly report increased carotenoid yields if an intermediate waiting step between PEF and solvent extraction was implemented.

Something similar was reported in other works such as Luengo et al. [38] and Martínez et al. [39] who noticed increased carotenoid yields if an intermediate waiting step between PEF and solvent extraction was implemented.

Therefore, the goal of the present work was to further explore this incubation step and its potential benefits to lipid extraction. More specifically, it was examined whether incubating the biomass after PEF-treatment had a positive effect on solvent consumption and how it affected the extraction mechanism. Two PEF-treatment energies were tested for this, 0.25 MJ/kg_dw_ and 1.5 MJ/kg_dw_. The PEF-treatment was always applied on wet microalgae directly after harvest, at 100 g_dw_/L concentrations. Extraction of lipids was performed either on freshly PEF-treated microalgae or on biomass that was incubated for 24 h after treatment. As model microalgae, *A. protothecoides* was used due to its proven high lipid content and documented previous success in cascade extraction after PEF-treatment [35]. Furthermore, a preliminary calculation of the energy demand for lipid extraction was performed in order to identify the key steps in need for improvement in the whole downstream process.

## Materials and methods

For all experiments wet and freshly harvested microalgae were used. All chemicals were of analytical grade.

### Microalgae cultivation and harvest

*A. protothecoides,* strain number 211-7a, was obtained from SAG, culture collection of algae Göttingen, Germany. The microalgae were cultivated in sterile conditions in autotrophic mode inside 25 L photobioreactors (PBR) in tris–phosphate (TP) medium for approximately 19 days. Detailed description of the PBR and the cultivation process is given in a previous work [40].

Harvest of the biomass was performed through centrifugation with a separator (STC 3–06–170, GEA Westphalia, Germany). The dewatered microalgae paste was re-suspended in a portion of the recovered supernatant in order to achieve a final cell dry weight of ~ 100 g/L. The exact final concentration was determined by overnight drying of known amounts of the final suspension and supernatant in a drying oven (Universalschrank Model U, Memmert, Germany) at 90 °C. For each harvest, portion of the harvested biomass was lyophilized (Alpha 1–4 LDplus, Christ) and stored in vacuum-sealed bags at −20 °C for further analysis.

### Pulsed electric fields (PEF) treatment and incubation

For PEF-treatment, a custom-made treatment chamber was utilized, capable of delivering uniform-field treatment, in a setup similar to the one used in previous works [35, 41]. The treatment chamber was composed of two parallel circular stainless-steel electrodes, 4 mm apart, separated by a polycarbonate housing. Treatment took place in continuous mode, with a flow rate of 0.1 mL/s and rectangular pulses of 1 μs duration and 40 kV/cm of electric field magnitude. The repetition rate of the pulses was either 0.5 Hz or 3 Hz resulting in input energies of 25 and 150 kJ/L, i.e., 0.25 MJ/kg_DW_ and 1.5 MJ/kg_DW_, respectively, as described in a previous work [35].

For incubation after PEF-treatment, the biomass was placed in polypropene falcons with screw cap (CELLSTAR® 50mL PP tubes, Greiner Bio-One, Frickenhausen, Germany). After flushing with N2, the samples were sealed and stored in the dark, without agitation, at 22 °C for 24 h.

### Yo-Pro uptake

Yo-Pro is a marker used as an indicator for cell membrane permeability. Permeabilized cells after PEF-treatment are therefore Yo-Pro positive. The microalgae suspension at 100 g/L concentration was diluted by a factor 1000 with its original filtered (0.2 µm) medium. From this diluted suspension, 1 mL was mixed with 10 µL of Yo-Pro (YO-PRO-1 Iodide 491/509, Invitrogen, Thermo Fisher Scientific) at 0.1 mM. The sample was incubated for 10 min at room temperature and in the dark. It was then diluted 1:5 before being analyzed with a flow cytometer (Attune NxT, Thermo Fisher Scientific with a 488 nm laser as excitation source). The emission fluorescence signal was collected with the green filter of the device (530/30).

### Monophasic lipid extraction

#### Monophasic lipid extraction kinetics

The kinetics of monophasic lipid extraction from *A. protothecoides* were studied using an upscaled version of the protocol for batch extractions from previous works [35, 41]. Freshly harvested wet biomass underwent PEF treatment at energy densities of 0.25 MJ/kg dw and 1.5 MJ/kg dw, at a concentration of 100 g/L. Immediately after treatment, 13.2 mL microalgae suspension were measured, dewatered through centrifugation at 10,000 × g for 10 min and re-suspended with ethanol in a glass flask (100-mL round bottom flask, Duran, Mainz, Germany). The rest of solvents were then added, so that in the end 71 mL ethanol and 29 mL hexane were used resulting in 75.6 mL of a monophasic ethanol:hexane:water co-solvent per 1 g dry biomass with composition 1:0.41:0.04 vol/vol/vol as in the batch process.

On designated time points, 10 min (0.17 h), 30 min (0.5 h), 120 min (2 h), 180 min (3 h), 240 min (4 h) and 1080 min (18 h), 12 mL sample, containing solvent and biomass, was removed in polypropylene falcon which was then centrifuged at 10.000 × g for 5 min. From the supernatant, 4 mL were removed in polypropylene falcons in duplicate. In these new falcons, 12 mL hexane and 1.9 mL distillated water were added. The samples were agitated for 5 min and centrifuged at 10,000 × g for another 5 min. From the upper phase, 10 mL were removed in pre-weighted glass tubes, evaporated under N_2_ and the lipid yields were calculated gravimetrically. The same exact process was repeated with biomass that was incubated for 24 h after PEF-treatment.

### Biphasic lipid extraction

Freshly harvested microalgae were treated with PEF at energy densities of 0.25 MJ/kg dw and 1.5 MJ/kg dw, at a concentration of 100 g/L. Immediately after treatment, 3 mL were measured in Teflon tubes and dewatered with centrifugation at 10,000 ×*g* for 5 min. The biomass pellet was re-suspended with 1.5 mL ethanol and 9 mL hexane. Overall, 37 mL of ethanol:hexane:water 1:6:0.4 vol/vol/vol solvent were used per 1 g of dry biomass. The system was biphasic with an ethanol–water lower phase and a hexane upper phase. After vortexing, extraction took place in the dark, room temperature and constant agitation for 24 h. The samples were then centrifuged at 10,000 ×*g* for 5 min. 7 mL were removed from the upper phase into pre-weighted glass tubes and evaporated under N_2_. The yields were determined gravimetrically. The same exact process was repeated with biomass that was incubated for 24 h after PEF-treatment.

**Fig. 1:**
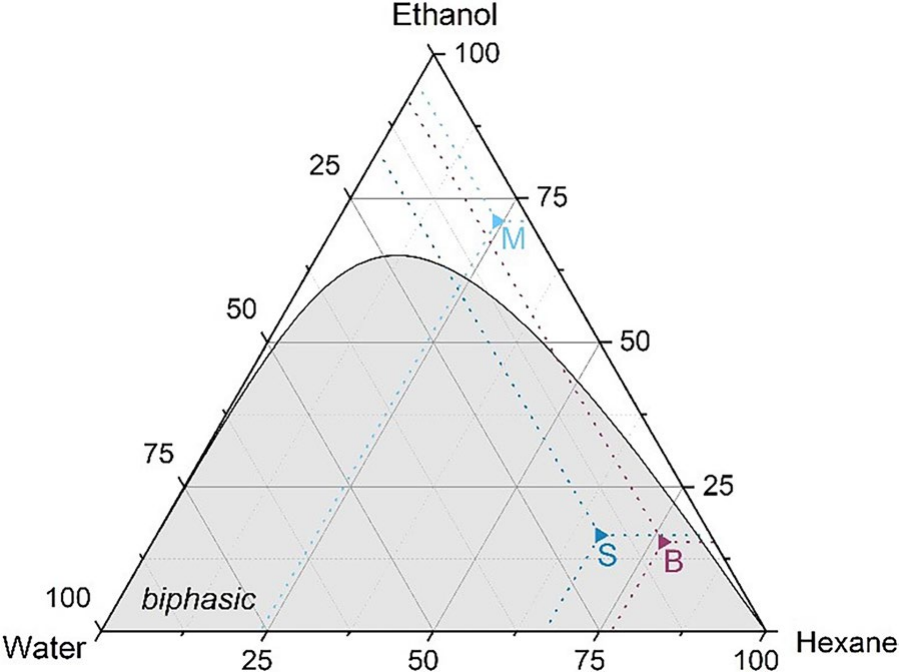
Ethanol:hexane:water phase diagram (% w/w/w). Data for phase separation limit were taken from [42]. Point M indicates the solvents fractions during extraction in the monophasic system and S the associated phase separation system. Point B indicates the solvents fractions in the biphasic extraction system

The monophasic and biphasic extraction systems are depicted in the ethanol:hexane:water tertiary plot in Fig. 1.

### Evaluation of total lipid content (adaptation of Kochert method)

As reference method for the evaluation of the total lipid content of the biomass, a chloroform:methanol protocol was followed, based on the Kochert method [43]. Freeze-dried biomass was subjected to bead milling at 30 Hz, 5 times for 15 s (Mixer mill, MM400, Retsch, Haan, Germany) and 0.1 g were measured in a precision balance in borosilicate glass tubes with screw caps (‘culture tubes’, 16/36/26 MP, Pyrex, England). In the next step, 2 mL of chloroform:methanol (2:1 vol/vol) were mixed with the biomass, vortexed and immediately centrifuged at 1800 × *g* for 4 min. After the centrifugation, the supernatant was removed and collected into a separate glass tube, 2mL of fresh solvent were added in the biomass pellet and the above process was repeated. In total, 7 mL of solvent were used, in four separate extraction steps (3 × 2 mL and 1 × 1 mL for the last step). In the glass tube with the collected solvent, 3 mL of HCl 0.1 N and 0.3 mL MgCl_2_ 0.5% were added and two distinct phases were formed. The lower phase along with the lipids was removed with a Pasteur pipette into pre-weighted glass tubes and evaporated under N_2_. The lipid yield was determined gravimetrically. All samples were performed in duplicates.

### Microscopy imaging on fixed and stained cells

Freshly harvested microalgae suspension, with a concentration equal to 1 g/L, were fixed in a bath of PBS (phosphate-buffered saline) solution containing 1% Triton for 4 min followed by 10 min in PBS with 2% formaldehyde. The cells were then washed twice using PBS before being stained with a 30 μg/mL Nile-Red solution. The staining was followed by two additional washing steps in PBS. The cells were then deposited on microscope slides which were sealed with varnish and kept at 4 °C. Observations were done using a spinning disk confocal microscope (Axio Observer Z.1) equipped with an objective Plan-Apochromat 63x/1.40 Oil DIC M27. Bright light pictures were acquired as well as fluorescence images in the GFP channel (excitation 488nm/emission 509 nm) and in the RFP channel (excitation 590nm / emission 612 nm).

### Evaluation of energy requirements

**Fig. 2:**
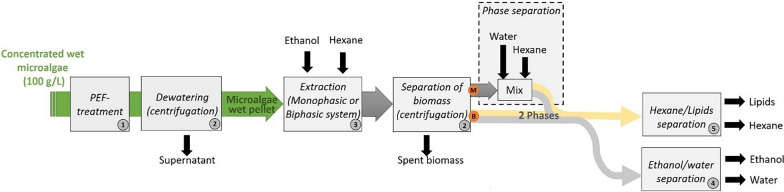
Flowchart of the examined lipid extraction processes. The letters M and B indicate the monophasic and biphasic processes, respectively. After the extraction phase itself, the addition of water and hexane is necessary in the monophasic approach to allow phase separation while this step is absent in the biphasic approach. The main energy consuming steps are indicated by the numbers 1 to 5 as described in the material and method

Τhe major energy consuming steps in the examined lipid extraction processes (monophasic or biphasic) are shown in Fig. 2 and include: (1) PEF-treatment; (2) centrifugation for dewatering after PEF-treatment and for removal of solvents from the spent biomass once extraction with ethanol:hexane is complete; (3) mixing during extraction; (4) ethanol–water separation; and (5) hexane–lipid separation. The last two steps constitute the solvent recycling segment of the process. Depending on the intensity of PEF-treatment (0.25 MJ/kg_DW_ and 1.5 MJ/kg_DW_), the inclusion of an incubation step after PEF and whether the extraction solvent was monophasic or biphasic, 6 different extraction pathways can emerge. The energy demand for each of them can be estimated from experimental data and a comparison between the different cases can be made.

All calculations have been made on the basis for the extraction of 1 L of lipids using upscaled experimental data. The equations for each major step are described below. Important parameters in the calculations are (a) the concentration of the microalgae suspension C_algae_, equal to 100 g/L; (b) the density of the *A. protothecoides* lipids reported equal to 0.91 kg/L [44]; and (c) the extraction efficiency η_ext_CDW_ (in percentage of CDW) which is given by experimental results for the different extraction system and is equal to the ratio of the extraction yield to the total lipid content as estimated by the Kochert method.

#### PEF-treatment of the biomass

The energy required for the extraction of 1 L of lipids with PEF application as pretreatment, ${\mathcal{E}}_{PEF}$[MJ/L_lipids_], is equal to:

1 $${\mathcal{E}}_{PEF}=\frac{{E}_{PEF}}{{\rho }_{lipids}* {\eta }_{ext\_CDW}},$$

where ${E}_{PEF}$ is the PEF input energy, i.e., in this study 0.25 or 1.5 MJ/kg_dw._

#### Centrifugation

The separation of microalgae either from excess water or from the extraction solvent can be accomplished through centrifugation, a process well established in the industry. Separators of all sizes and shapes exist [45]. At industrial level, the energy demand for centrifugation could be assumed equal to Ε_centrifugation_ = 0.7 kWh/m^3^ or 0.00252 MJ/L of feed [46] meaning that for 1 L of lipids the centrifugation energy ${\mathcal{E}}_{cent}$ [MJ/L_lipids_] would be calculated as:

2 $${\mathcal{E}}_{cent}=\frac{{E}_{centrifugation}}{{C}_{algae }*{\rho }_{lipids}*{\eta }_{ext\_CDW}}.$$

#### Mixing energy demand

Mixing would be required during the lipid extraction to ensure a homogeneous and constant contact of the solvents and the microalgae. For the calculation of energy demand, the equation given by Halim et al. [47] was adapted. For 1L of lipids, the ${\mathcal{E}}_{mixing}$ [MJ/L_lipids_] is equal to:

3 $${\mathcal{E}}_{mixing}=\frac{{I}_{mixing{*t}_{mixing }*{ V}_{total}}}{{\rho }_{lipids}{*\eta }_{ext}},$$

where I_mixing_, a parameter representing the mixing intensity and equal to 0.001 MJ/s·m^3^ as estimated by Martin et al. [48], t_mixing_ the duration extraction, i.e., 86,400 s (24 h), V_total_ the total volume to mix.

#### Ethanol/water separation (ethanol recycling)

High purity ethanol (99% wt) is required for lipid extraction which has to be recovered and reused. At the end of the lipid extraction, large volumes of ethanol remain mixed with water. Their separation however, is particularly challenging due to the formation of azeotrope point at ~ 95.6% wt ethanol, beyond which they cannot further separate without more advanced techniques. A precise theoretical calculation of the energy requirements was beyond the scope of this paper. Most of relevant studies conducted so far, examined the production of anhydrous ethanol from fermentation, where ethanol is initially present in low concentrations (~ 5–10% wt EtOH). Based on an extensive review by Vane [49] a value of Ε_ethanol_ = 2.34 MJ/kg_EtOH_ is required to produce 99% wt ethanol starting from a 10% wt ethanol aqueous solution. In the monophasic and biphasic extraction processes proposed in the present article, pure ethanol needs to be recovered from 51% wt and 67% wt ethanol aqueous solution, respectively. Nevertheless, the value proposed by Vane was taken for the calculations due to the lack of more precise data in the literature, with the conscious risk of potentially overestimating the energy requirement:

4 $${\mathcal{E}}_{\text{EtOH}}=\frac{{\rho }_{lipids}*{V}_{lipids}}{{\eta }_{ext\_CDW}}*{V}_{EtOH}*{\rho }_{EtOH}*{E}_{EtOH},$$

where V_EtOH_ is the total volume of the solvent used in extraction and ρ_ΕτΟΗ_ the density of EtOH, equal to 0.789 kg/L.

#### Hexane/lipid separation (hexane recycling)

This step has the double function of separating the lipids as a product and recovering hexane for reuse. Hexane distillation is a process favored by the industry mainly due to the solvent’s narrow boiling point [50]. The evaporation energy of hexane can be calculated theoretically as seen in Halim et al. [47]:

5 $${{\mathcal{E}}_{hex }=\uplambda }_{hex}{*m}_{hex}{*n}_{heat\, recovery}={\lambda }_{hex}*\frac{{\rho }_{lipids}*{V}_{lipids}}{{\eta }_{ex{t}_{CDW}}}*{\rho }_{hex}*{V}_{hex}*{\eta }_{heat\, recovery,}$$

where λ is the latent heat of vaporization of hexane (0.34 MJ/kg_hexane_), V_hex_ the total volume of the solvent used in the extraction and n_heat recovery_ the efficiency of recovering the heat energy from solvent evaporation (assumed 70%[47]).

#### Energy consumption ratio (ECR)

The sustainability of the downstream process from an energy point of view was evaluated by estimating the Energy Consumption Ratio (ECR), i.e., the energy consumed during processing divided by the heating value of the obtained product (lipids in this case) [51]. As a rule, ECR should be at least lower than 1 to indicate a positive balance, something particularly important for energy applications:

6 $$ECR=\frac{{\mathcal{E}}_{D}}{{\mathcal{E}}_{L}}=\frac{{\mathcal{E}}_{PEF}+{\mathcal{E}}_{centrifugation}+{\mathcal{E}}_{EtOH}+{\mathcal{E}}_{hex}}{{\mathcal{E}}_{L}},$$

where ${\mathcal{E}}_{D}$ the energy consumed during the downstream process, equal to the sum of the energy of all individual steps from the previous section and ${\mathcal{E}}_{L}$ the energy of extracted lipids. The energy content of microalgae lipids is reported to be equal to 38.3 MJ/kg [52]. According to Batista et al., the density of *A. protothecoides* lipids is equal to ~ 0.92 kg/L at 25 °C [44], and therefore 1 L of *A. protothecoides* lipids would have a ${\mathcal{E}}_{L}$ equal to 35.2 MJ.

### Data fitting

The non-linear fitting of the experimental data was performed using the commercial software Origin. For each fit, the coefficient of determination (COD) or R-square is reported (Eq. 7):

7 $${R}^{2}=1- \frac{RSS}{TSS},$$

where RSS the residual sum of square, i.e., the portion that is not explained by the regression model and TSS the total sum of square.

## Results

### Yo-Pro uptake of *A. protothecoides* after PEF-treatment

**Fig. 3:**
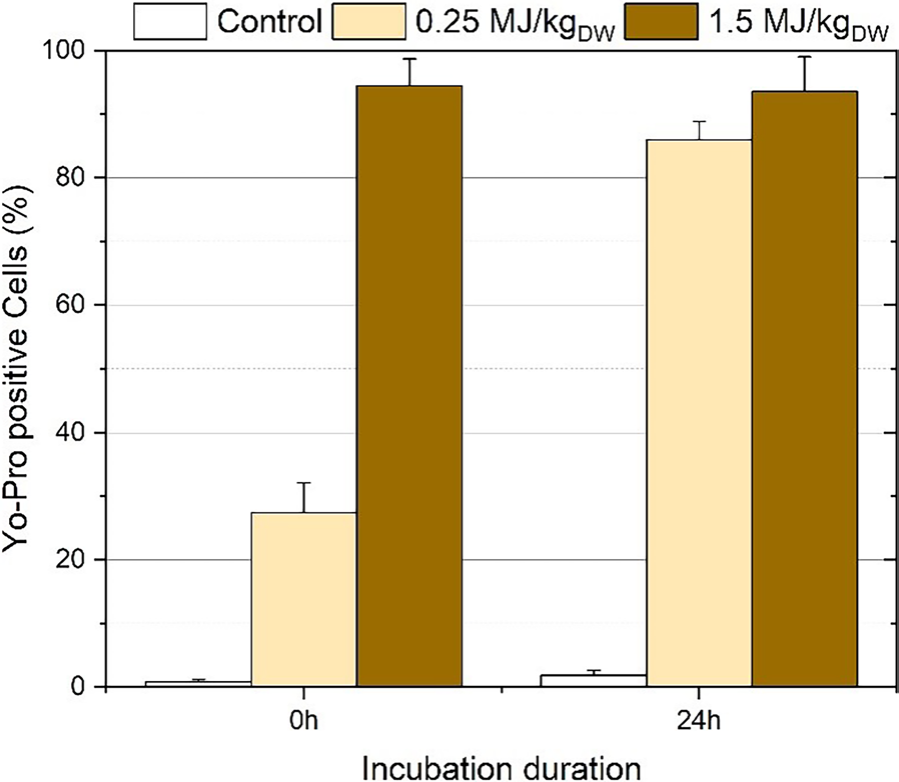
Fraction of Yo-Pro positive cells (*A. protothecoides*) directly or 24 h after PEF-treatment. Results are the average and standard deviation of two independent experiments

Yo-Pro uptake from the microalgae cells is an indicator of the degree of permeabilization of the cell’s plasma membrane. Microalgae were stained after PEF, either immediately after treatment or after a 24 h incubation step. The results can be seen in Fig. 3.

About 1% of untreated microalgae were stained by Yo-Pro, and this percentage barely increased after the incubation of 24 h, demonstrating that *A. protothecoides* could withstand the incubation without any loss of membrane permeability. On the contrary, immediately after PEF-treatment of biomass with an energy of 1.5 MJ/kg_dw_, 94% of the cells were stained with Yo-Pro, indicating an immediate permeabilization of almost all the cell population without any need for incubation. In case the energy of the PEF-treatment was reduced to 0.25MJ/kg_DW_, 27% of the cells were stained with Yo-Pro immediately after the treatment, while incubating the biomass after PEF-treatment increased the percentage of stained cells to 86%. These results are in agreement with the tendency observed in a previous study [35]. They illustrate the evolution of the biomass during incubation after PEF-treatment and suggest a gradual harmonization over time between microalgae treated with the two different energies: a treatment energy as low as 0.25MJ/kg_DW_ being compensated by a 24-h waiting time.

### Monophasic lipid extraction kinetics

**Fig. 4:**
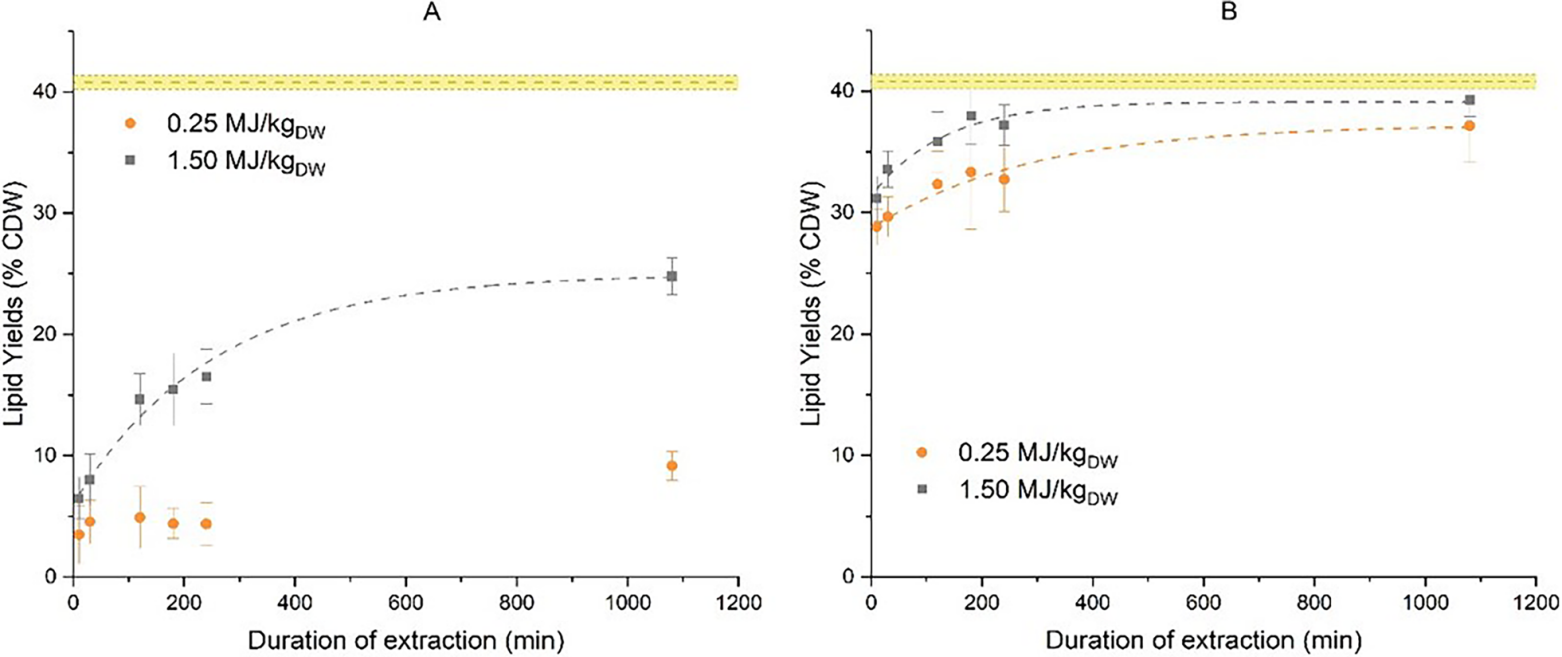
Kinetics of lipid extraction from *A. protothecoides* using monophasic ethanol:hexane:water, 1:0.41:0.04 vol/vol/vol, co-solvent system with 1g:78mL biomass:solvent ratio after PEF-treatment (**A**) without any incubation or (**B**) with a 24 h of incubation after PEF-treatment (**B**). No kinetics extraction was performed from untreated biomass given the insignificant yields (lipid yield from extraction performed on small volumes of control biomass were in the range of 0.8–1.81%). The dashed lines represent the non-linear fitting performed by Origin software using the one-phase exponential decay function with a time constant parameter expressed as C(t) = C_w_ + C_D_(1- exp(-k_d_t)) as detailed in paragraph 3.2. The straight line in the yellow zone indicate the total lipid content of the biomass as determined by chloroform:methanol extraction. Results are the average ± std from three independent cultivations with internal duplicates

As demonstrated in previous studies [35, 41] a monophasic ethanol:hexane:water co-solvent system, 1:0.41:0.04 vol/vol/vol, using 78 mL of solvent per 1 g dry weight, was effective for lipid extraction from wet microalgae after PEF-treatment. Using this protocol, a study of the kinetics of the extraction was performed with the goal of gaining a greater insight on the extraction mechanisms and on the effect of incubation. The yields at 6 different time points were measured gravimetrically. Once again, two PEF-treatment energies were applied, 0.25 MJ/kg_dw_ and 1.5 MJ/kg_dw_, with or without a 24-h incubation between PEF-treatment and the start of extraction. No kinetics of extraction was performed on untreated biomass since the ineffectiveness of lipid extraction in the absence of any pretreatment with this microalga and solvent system was already well documented [35, 41]. However, extractions were still conducted on small volumes of control biomass to verify the necessity of a pretreatment method and were in the range of 0.8–1.81% dry weight. The results can be seen in Fig. 4:

In case the extraction is performed directly after PEF-treatment (Fig. 4A), the lipid yield obtained from biomass treated with 0.25 MJ/kg_dw_ and extracted for 10 min is 3.5% dry weight only. After 240 min extraction, the yield barely increased to 4.4%. At the end of the extraction (1080 min), the final lipid yield was 9.2% dry weight. For microalgae treated with 1.5 MJ/kg_dw_, the yield at 10 min was also low, i.e., 6.5% dry weight. At the end of the extraction (1080 min) there was a further increase of the yield, up to 24.8% dry weight.

The kinetics of lipid extraction from biomass that was incubated after PEF-treated are displayed in Fig. 4B. For both treatment energies, at 10 min the lipid yields were significantly increased (28.8% and 31.2% dry weight for the PEF-treatment energies of 0.25 MJ/kg_dw_ and 1.5 MJ/kg_dw_, respectively). Over the course of 240 min extraction, the lipid yields were increased το 32.7% for 0.25 MJ/kg_dw_ and 37.2% dry weight for 1.5 MJ/kg_dw_. The yields then remained relatively stable until the end of extraction (1080 min), with final yields 37.2% and 39.3% for 0.25 MJ/kg_dw_ and 1.5 MJ/kg_dw_, respectively, indicating saturation. The total lipid content of the biomass was evaluated with chloroform:methanol extraction (Kochert method) from freeze-dried, bead-milled microalgae and found equal to 40.8 ± 0.6% dry weight, indicating a high efficiency of extraction from incubated biomass.

It therefore appears that incorporating an incubation step in the lipid extraction not only allows for a reduction of the energy of PEF-treatment, but also leads to a significant acceleration of the extraction process as well, especially visible when examining the high yields after only 10 min of extraction. These results also bring valuable insights on the lipid extraction mechanism. At conditions in which complete permeabilization of the microalgae cells is certain (such as 1.5 MJ/kg_dw_, as seen in Fig. 3), the lipid extraction is a slow process when extraction is performed directly after the PEF-treatment, i.e., without incubation. Therefore, it seems that in such case, the extraction is mainly driven by slow processes such as diffusion, a plausible assumption considering that the overall microalgae cell structure remains intact after PEF-treatment, unlike with other more intense pretreatment methods such as bead milling or high pressure homogenization. However, the extraction rate after incubation is quite different, with a fast lipid recovery followed by a slow, but still noticeable, further increase of the yields.

The observed lipid extraction behavior could be roughly divided in different zones. An initial, rapid jump-like increase in extraction yields followed by an slower increase due to diffusion, which is typical for description of extraction processes [53]. This two-step behavior, a rapid solvation or ‘washing’ and a slower diffusion, is reminiscent of the empirical Patricelli model [54, 55]. The mathematical transcription of this model is given in the following equation:

9 $${C (t)}={C}_{w}\left(1-exp\left(-{k}_{w}t\right)\right)+{C}_{d}\left(1-exp\left(-{k}_{d}t\right)\right),$$

where C(t) is the lipid yield at any time, C_w_ is the lipid yield at equilibrium for the fast washing step, C_d_ is the lipid yield at equilibrium for the diffusion step, k_w_ the kinetic coefficient for the washing step, k_d_ the kinetic coefficient for the diffusion step and t is the time. However, when the microalgae are stirred in a solvent mixture, the easily accessible lipids might dissolve so fast that we do not have the time resolution to measure the kinetics of the washing step. Therefore, only the kinetics of the diffusion step can be resolved. [56]. This seems to be the case in the experimental data shown in Fig. 4, where within 10 min this initial washing step has already been saturated. Under the assumption that before the first sampling at that time point, the washing step is finished, a more simplified equation can be used to fit the experimental data:

10 $${C(t)}={C}_{w}+{C}_{d}\left(1-exp\left(-{k}_{d}t\right)\right).$$

Non-linear curve fitting of the lipid extraction yields from this experiment was performed with the data analysis software Origin, using Eq. (10) and results are given in Table 1 and the fitting curves displayed in Fig. 4.

**Table 1:** Coefficients and statistical parameters of lipid extraction modeling from *A. protothecoides* after PEF-treatment, using the Patricelli model and the equation ${C(t)}={C}_{w}+{C}_{d}\left(1-exp\left(-t/t_1\right)\right)$

Equation	${C(t)}={C}_{w}+{C}_{d}\left(1-exp\left(-t/t_1\right)\right)$			
Plot	No incubation		24-h incubation	
	0.25 MJ/kg_dw_	1.5 MJ/kg_dw_	0.25 MJ/kg_dw_	1.5 MJ/kg_dw_
C_W_, % CDW	4.94 ± 0.18	6.00 ± 1.94	28.70 ± 1.82	31.36 ± 1.51
C_D_, % CDW	N/A	18.98 ± 1.08	8.54 ± 0.91	7.76 ± 0.90
t_1_, min	N/A	251.6 ± 40.7	287.9 ± 77.4	129.7 ± 41.0
R^2^ (COD)	N/A	0.98	0.96	0.94

Evaluation of the fitting, can be done by examining the calculated values of coefficient of determination R^2^. As can be seen in Table 1, the Patricelli model was a proper fit with the exception of 0.25 MJ/kg_dw_ without incubation. A possible explanation for this could be the ineffective lipid extraction at this condition due to the incomplete permeabilization of the cells and that more time was required for saturation, as seen in Fig. 3. For the rest of conditions, R^2^ was higher than 0.9 indicating a proper fit.

The lipid extraction mechanism from microalgae after PEF-treatment is largely unknown, to an extent due to the lack of information of the effect of PEF-treatment on the cell wall (in contrast to its well documented effect on the cell membrane). In a review, Allaf and colleagues note that when solvent extraction is performed on plant material [57] the factor limiting the extraction is the internal solute-to-solvent transfer in the pores of the solid matrix. Since the main obstacle to this transfer in microalgae is the cell wall, one potential explanation to the extraction kinetics presented above, could be that cell wall degradation is taking place during incubation due to the release of intracellular enzymes after PEF-treatment as theorized by Martínez et al. [22]. That could potentially explain both the accelerated extraction kinetics after PEF-treatment and the discrepancy between Yo-Pro uptake and lipid yields immediately after PEF, indicating the cell wall as the main barrier to lipid extraction.

### Microscopy examination of microalgae lipids distribution

**Fig. 5:**
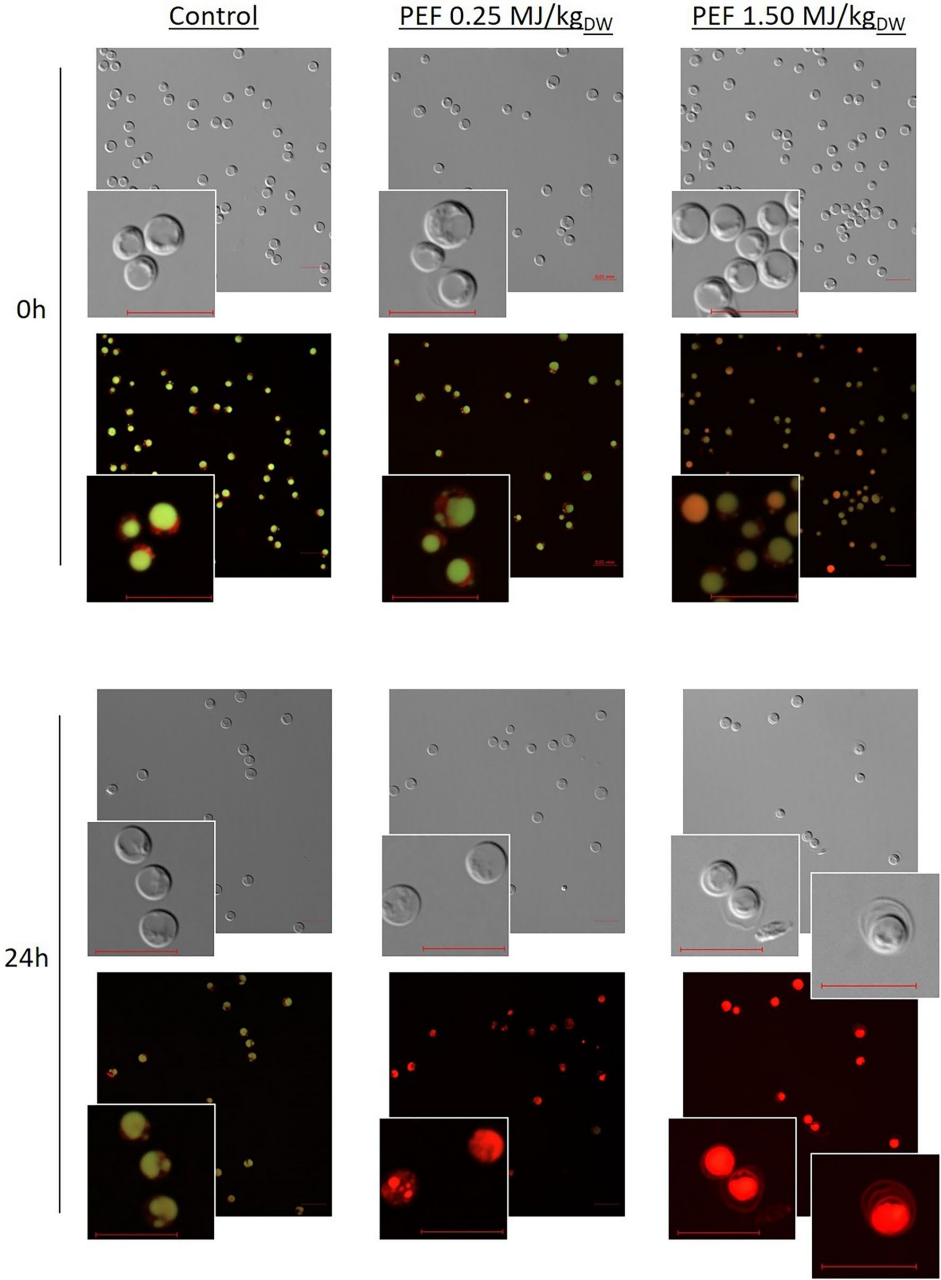
Observation of lipid distributions after PEF-treatment. Cells were fixed either immediately or 24 h after PEF treatment and stained with Nile-Red. The images are the bright field and the overlay of GFP (green) and RFP (red) channels. The bars are 10 μm. A The emission shifting from both GFP and RFP to mostly FRP could indicate an increase of polarity and mixture of lipids with more polar cell components

In order to explore the potential evolution of the microalgae lipids during this incubation, microalgae cells were fixed immediately or 24 h after PEF-treatment, stained with Nile-Red and observed using fluorescent microscopy. Images displayed on Fig. 5 are the bright field pictures and the overlay of the GFP (green) and RFP (red) channels. On the control cells immediately after harvest, the lipids were clearly visible as one main large droplet in each cell, both in the GFP and RFP channel (i.e., yellow on the overlay), while a distinct red signal was emitted from chloroplast. Similar patterns are observed after 24 h of incubation, indicating that the cell morphology was stable in the absence of PEF-treatment. Those patterns could also be seen immediately after the PEF-treatment of low energy, i.e., 0.25 MJ/kg_DW_, while 24 h after the treatment, signal was mainly coming from the RFP channel with no distinct structure visible anymore. In the case of the high intensity PEF-treatment, i.e., 1.5 MJ/kg_DW_, the disappearance of cells structure was visible immediately after the PEF-treatment and fluorescence was distributed homogeneously inside the whole cells. On the fluorescence merge image, the color appears sometimes yellow, sometimes red, indicating either emission in both GFP and RFP channels or mostly RFP. After 24 h of incubation, all cells displayed a homogeneous signal distributed in the whole cells, and emitting predominantly in the RFP channel. Additionally, cell walls appeared to be detaching from the cells. The shift of emission from both GFP and RFP channels to mostly the RFP channel indicates a shift of emission of fluorescence of Nile-Red to the higher wavelength which corresponds to an increase in the polarity of the environment of Nile-Red. It therefore appears that the lipids are not anymore encapsulated in the well spatially defined droplets, but are mixed with other components of the microalgae, creating therefore a more polar environment around them.

### Biphasic lipid extraction

Since incubating the microalgae after PEF-treatment was shown to have a drastic effect on the kinetics of the extraction and on the polarity of the lipids, it was examined whether it could be utilized for solvent reduction. A different extraction strategy was thus followed, by applying a biphasic lipid extraction system. The assumption behind was that since the lipids seemed to be in a more polar environment after PEF-treatment (as indicated in Fig. 5), then their solubility in a solely polar solvent such as ethanol might increase.

The hypothesized mechanism of this system is that extraction is taking place in the polar phase with the lipids then transitioning over to the hexane phase, both phases being permanently in contact thanks to a constant agitation on an orbital shaker. With this approach the extracting polar phase in which the biomass is dissolved is permanently lipid-free, therefore maximizing the lipid-gradient between the cells inside and the extracting phase. As an additional benefit, extraction in a biphasic system enables to bypass the phase separation step which requires additional solvents, allowing for further reduction of solvent consumption.

**Fig. 6:**
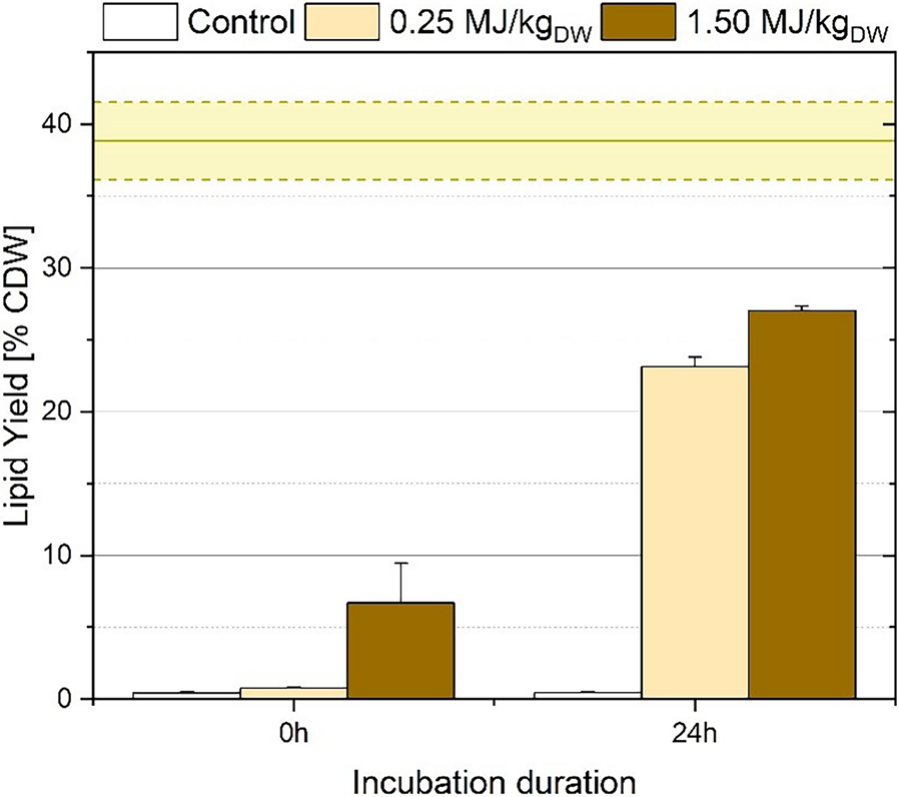
Biphasic lipid extraction from wet *A. protothecoides* after PEF-treatment with 0.25 MJ/kg_dw_ or 1.5 MJ/kg_dw_. The solvent mixture used was a biphasic ethanol:hexane:water co-solvent system 1:6:0.4 vol/vol/vol with a ratio of 37 mL per 1 g of dry biomass. The straight line in the yellow zone indicate the total lipid content of the biomass as determined by chloroform:methanol extraction. Extraction took place either immediately or after 24-h incubation after PEF-treatment. Results are the average + std of three independent experiments

The approach proposed above was tested on wet *A. protothecoides* subjected to PEF-treatment, eventually followed by a 24-h incubation step at inert conditions. The results are presented in Fig. 6.

Immediately after PEF-treatment, the lipid yields from untreated biomass and 0.25 MJ/kg_dw_ were negligible. Extraction from 1.5 MJ/kg_dw_ was also not successful, with yields limited to 5% dry weight. Incubating the biomass after PEF greatly improved the results, however. The yields after PEF treatment with 0.25 MJ/kg_dw_ and 1.5 MJ/kg_dw_ were increased to 23% and 27% dry weight, respectively, i.e., 59% and 69% of total evaluated lipids. The biphasic extraction approach, while completely ineffective on freshly treated microalgae, was highly effective on incubated biomass.

Adaption of the biphasic system offers some very important advantages for lipid extraction. Apart from the solvent amounts spared during extraction, no further hexane or water is required for phase separation. Moreover, the significant reduction of ethanol is of high economic value given its difficulty to be recycled from water. This is further discussed in the next section.

### Energy evaluation of lipid extraction

In the previous sections, lipid extraction at laboratory-scale from microalgae after PEF-treatment was examined. Here, an early estimation of the energy demand of downstream processing with a focus on the lipid extraction along with the solvent recycling is performed. The energy demand for the other biorefinery segments, such as cultivation, harvest or product upgrading were not included in this evaluation. The goal was to have a first impression of the energy required for the extraction of 1 L of lipids from *A. protothecoides* after PEF-treatment which could provide useful insights on the feasibility of the process and detect the most critical steps of the process.

Equations (1–5) were used for calculation of the energy demand for each significant downstream process while the energy consumption ratio (ECR) was calculated using Eq. (6). The experimental data from Figs. 4 and 6 were used for the estimation of the extraction efficiency. Six separate pathways were formulated based on the PEF-treatment energy, the inclusion of incubation, the extraction efficiency and the type of extraction system (monophasic or biphasic). The results of the calculations are summarized in Table 2.

**Table 2:** Energy demand of the lipid extraction process for 1 L lipid calculated from different routes with varying experimental data from Sect. 3.2 and 3.4

Process	Route 1	Route 2	Route 3	Route 4	Route 5	Route 6
	Monophasic				Biphasic	
Parameters						
PEF treatment (MJ/kg_Dw_)	0.25	0.25	1.5	1.5	0.25	1.5
Incubation (h)	–	24	–	24	24	24
Extraction (h)	18	18	18	18	18	18
Ethanol (L)	537	132	199	125	20	17
Hexane (L)	2832	697	1050	661	119	102
Extraction efficiency (%)	22.4	91	60.4	96	59	69
Energy demand of the different steps of lipid-extraction (MJ/L_lipids_)						
PEF-treatment	2.5	0.61	5.48	3.5	0.95	4.9
Removal of water fraction	0.25	0.02	0.09	0.06	0.10	0.08
Mixing during lipid extraction	49.9	12.3	18.5	11.6	9.1	7.8
Separation of solvent from spent biomass	1.9	0.5	0.7	0.5	0.4	0.3
Ethanol recycling	995	245	364	232	35	30
Hexane recycling	439	108	160	102	19	16
Total	1489	366	549	349	65	59
Energy consumption ratio (–)	42.3	10.4	15.6	9.9	1.8	1.6

The extraction efficiencies are taken from experimental data from Figs. 4 and 6

Many interesting insights can be gained from Table 2. For each pathway, the ECR was higher than 1, meaning that more energy is consumed for lipid extraction than the energy they contain, something prohibitive, at least for energy applications. This is mostly due to the recycling of the organic solvents which represent the bulk of energy demand, ethanol in particular. Indeed, in the monophasic-system approach, even for incubated microalgae treated with 1.5 MJ/kg_dw_ (Route 4), 232 and 102 MJ/L_lipids_ are required for ethanol and hexane recycling. By comparison, the energy required for the rest of the steps, such as PEF-treatment energy (3.5 MJ/L_lipids_), seems negligible by comparison and their optimization less important at this stage. As mentioned before, this is due to the formation of an azeotrope between ethanol and water which renders conventional separation techniques ineffective. Unfortunately, reduction of solvent in this monophasic approach (i.e., increase of the biomass:solvent ratio) was shown to be inefficient in unpublished results, with dramatic reduction of yields.

The critical role of solvent volume and especially the role of ethanol can, however, be greatly minimized using the biphasic approach with solvent reduction. The monophasic extraction protocols demand large amounts of ethanol and hexane. Even in conditions with the highest extraction efficiency, by incubating the biomass after PEF-treatment with 1.5 MJ/kg_dw_, still 125 mL ethanol and 51 mL hexane were required. The solvent consumption was further compounded by adding a phase separation step to isolate the lipids and the addition of another 610 mL hexane. In contrast, the biphasic extraction in the same conditions (1.5 MJ/kg_dw_ and incubating the biomass), require only 17 mL and 102 mL of ethanol and hexane, respectively.

As a result the ECR of the biphasic extraction was lower almost by a factor 10 compared to the monophasic one, even though the extraction was not as efficient (69% vs 96% for the biphasic and monophasic extraction, respectively, when a PEF-treatment of 1.5 MJ/kg_dw_ and an incubation of 24 h). This also highlights that the type and volume of solvent can overshadow the effectiveness of the extraction.

To render the process more commercially feasible, it is imperative to focus first on the matter of the solvents. The extraction efficiency of the biphasic method could be further improved which would lead to reduced solvent consumption. The hexane could potentially be reused several times without evaporation since the amount of lipids dissolved was far from saturation. Another option could be the replacement of ethanol with a different alcohol, one that does not form azeotrope with water. As demonstration of potential benefits, it could be assumed that an equal solvent amount of methanol would result to similar extraction efficiencies with the observed ethanol ones. Replacing ethanol with methanol in the biphasic route 6 and using Eq. (5) but with respective methanol data, could further reduce the ECR to 1.16. However, the efficiency of methanol/hexane extraction after PEF-treatment needs to first be experimentally proven and monitored.

## Conclusion

In this work, the effect of incubating microalgae after PEF-treatment prior to lipid extraction was examined. Beyond its already known positive impact on lipid yields and reduction of treatment energy, it was found that the extraction kinetics were significantly improved after incubation, with 10 min being sufficient to extract the majority of the lipids. The evolution of the biomass during incubation also allowed for an (otherwise ineffective on fresh microalgae) mildly successful application of a biphasic ethanol:hexane:water 1:6:0.4 vol/vol/vol solvent. This minimization of ethanol volume demand led to an up to 90% reduction of the energy demand for the process, improving its future commercial prospects.

## Data Availability

No datasets were generated or analyzed during the current study.
